# Review on the prevalence of diabetes and risk factors and situation of disease management in floating population in China

**DOI:** 10.1186/s41256-017-0053-8

**Published:** 2017-11-08

**Authors:** Kaiyi Han, Jingjing Yao, Xiao Yin, Mei Zhao, Qiang Sun

**Affiliations:** 1 School of Health Care management, Key Lab of Health Economics and Policy Research, NHFPC, Shandong University, No.44, Culture west road, Lixia District, Jinan, Shandong Province 250012 China; 2 Ji’nan Central Hospital, NO.105, Jiefang Road, Ji’nan, Shandong Province 250013 China; 3 Department of Health Administration, Brooks College of Health, University of North Florida, UNF Drive, Jacksonville, FL 32224 USA

**Keywords:** Diabetes, The floating population, Risk factor, Disease management

## Abstract

**Objective:**

To give a comprehensive and basic understanding of diabetes and its risk factors in floating people in China.

**Method:**

We use "(diabetes or type 2 diabetes or chronic disease) and (floating population or employed floating population)" as the key words to search in the China academic literature database (CNKI), Wan Fang database, PubMed and Web of Science for relevant literature and extract the data about the prevalence of diabetes, relevant risk factors and disease management of the floating population in China.

**Result:**

Twenty-one literatures are entered into analysis finally, one is English and the rest are Chinese. According to the national survey, the prevalence rate of diabetes in floating population in China was 5. 1% (95%CI, 4.9% - 5.3%), which is lower than that of the general population(11.6%,95%CI, 11.3%–11.8%),and is consistent with the results of the local surveys. The comparison result between the prevalence of floating population and that of local population in each region differs in local surveys. In addition, the prevalence of male floating population is lower than that of the female population. Finally, as the age of the population rises, so does the prevalence of diabetes. As for the risk factors of chronical diseases, the overweight rate in every region is similar but the obesity rate differs in different regions (Ningxia,26.0%;Xiangshan,14.0%), and the obesity rate of the floating population is less than half of that of the general people(4.7%,11.9%). The awareness rate, treatment rate and control rate in the floating patients differ in the regional researches, but they all can’t meet the goals set by the local health departments.

**Conclusion:**

Compared with the general population, the prevalence of diabetes in the floating population are lower. However, Considering the growing population number and the poor disease management of the floating patients, the potential threat brought by the diabetes in floating population is imponderable. The government should establish the national surveillance system of diabetes for the floating population, strengthen the construction of the primary medical institutions, and optimize the existing funding system.

## Background

The floating population is a special social group formed in China’s social and economic development in recent decades, mainly refers to the adults at the childbearing age who leave their domicile for the purpose of making a living [1]. According to the latest statistics, the number of floating population in China has reached 247 million [2]. Due to the low level of education, poor living conditions and other reasons, the health status of the population is poor, and the prevalence of chronic diseases is very high [3]. Diabetes, as one of the chronic diseases, causes the pain, disability and high medical expenses which not only seriously affect the quality of patients’ life, but also lead to serious direct and indirect economic loss. Meanwhile, there exists vast disparities of economic level, education, health insurance, health services utilization between the floating population and local residents, and the data about prevalence of diabetes and its risk factors in floating population is lacking. The review was designed to focus on the prevalence of diabetes and its risk factors and the disease management in the floating population.

## Methods

### Literature retrieval

A systematic search was performed using China academic literature (CNKI), Wan Fang, PubMed and Web of Science databases across the period 1990–2016 to identify relevant researches. Search terms used either singularly or in combination were ‘diabetes’, ‘type 2 diabetes’, ‘chronic disease’, ‘floating population’, ‘migrant worker’ and ‘employed floating population’ in the thesaurus and index lists of the relevant databases. Also ‘free text’ words were used to supplement the search terms [medical subject heading (MeSH) search terms in the case of Medline]. Manual searches of the bibliographies of searched articles and reviews in the field were also conducted.

#### Information extraction and literature quality evaluation

The exclusion criteria:(1) review article, questionnaire reliability and validity research; (2) non-Chinese mainland floating population; (3) the lack of related data. The information table was designed by the research team, and the information was extracted by 2 researchers. The opinions were decided by the task group when the opinions were extracted. Literature evaluation criteria recommended by the Agency for Healthcare Research and Quality (AHRQ) in the United States were used to evaluate the cross-sectional study quality [4]. The scale consisted of 11 items, including subjects, selection, research, quality control and data processing, using the “yes”, “no” and “unclear” as answers. The quality evaluation was conducted independently by 2 researchers, and the decision was made by the senior researchers when differences occurred.

## Results

### Incorporation of literature

The literature numbers of the initial retrieval in the CNKI, Wan Fang data, PubMed, and the Web of Science were 98, 75, 14 and 2, respectively. The final sample included 21. The subjects of the studies were distributed in different regions in China, different level units such as the provinces, municipalities and counties were included.

### Literature quality evaluation

As can been seen in Table 1, all of the studies were conducted after 2010, and the location of the study was distributed in different provinces, autonomous regions, and municipalities in mainland China. Among these studies 3 studies conducted in the district administrative region, 7 studies in the administrative regions of city, 11 studies in the administrative regions of the province and 1 study in the whole country. The sample size of those studies ranged from 302 to 49,704. The scores judged by the cross-sectional study quality evaluation criteria recommended by AHRQ showed that most of them have relatively good research quality.

**Table 1:** Quality evaluation of the included literature

First author	Published year	Sample size	Study area	Research field type	quality sore
Yufang Bi	2016	48 704	all 31 provinces, autonomous regions, and municipalities in mainland China	nationwide	7
Ling Chen	2015	302	Bei Hai City, Guang Xi province	municipal	7
Zhenwang Fu	2014	600	Hai Nan province	provincial	9
Tianjing He	2016	1724	Hu Bei province	provincial	5
Kui Ji	2015	2371	Si Chuan province	provincial	5
Donghui Jin	2015	2098	Hu Nan province	provincial	8
Hua Li	2015	2378	He Bei province	provincial	6
Chunxia Liu	2015	303	Qin Huangdao city, He Bei province	municipal	5
Lixia Ma	2014	610	Ning Xia province	provincial	5
Xiuyun Sun	2011	801	Chongwen district, Beijing	district	2
Wenyun Wang	2016	600	Xiangshan city, Zhejiang province	municipal	5
Xiaofei wu	2013	300	Kashi city, Xingjiang province	municipal	4
Kaixu Xie	2013	1800	Tonxiang city, Zhejiang province	municipal	6
Wei Yan	2015	1475	Jiangxi province	provincial	4
Xin Yao	2014	1500	Inner Mongolia province	provincial	5
Chuanhua Yu	2016	1800	Hu Bei province	provincial	5
Yine Zhang	2016	610	Ning Xia province	provincial	5
Xiaohong Zhou	2015	303	Xia Chen district, Hangzhou city, Zhejiang province	district	5
Yingzhe Huang	2013	306	Binyang county, Guangxi province	district	6
Lei Qiao	2010	880	Beijing	municipal	6
Xin Meng	2015	1493	Ji Ling province	provincial	9

### The prevalence of diabetes in the floating population and comparison with general population

**Fig. 1:**
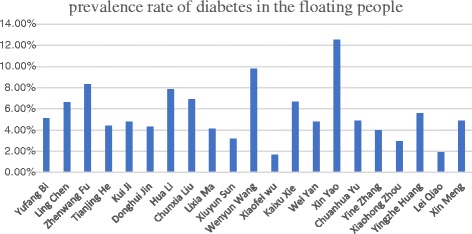
The prevalence rate of each study

According to the latest national survey, the prevalence of diabetes in Chinese adults is about 11.6% (95%CI, 11.3–11.8) [3]. The prevalence of diabetes in the floating population is 5.1% (95%CI,4.9–5.3) [4]. By comparing the findings from the studies of the provincial, municipal, and county level, we find that except for a few studies, the majority of studies show that the prevalence of diabetes among the floating population is significantly lower than that of the general population in Hainan province and Xiangshan, which is an administer county in Zhejiang province, where the prevalence rates in the floating population over 18 years old are both 9.8%, which are close to the national population level [3, 5]. Meanwhile, in Inner Mongolia**,** the prevalence rate of the floating population is higher than the national general population level [6]. While the findings of most studies fluctuate around the prevalence rate of the nationwide floating population, some studies indicate that prevalence rates in some district are in very low level. For example, the prevalence rate is 1.7% in Kashi, Xinjiang Province [7]. while the rate in one district in Beijing is only 1.9% [8].However, no study has done further analysis on the difference of the prevalence rate of the floating population in different regions. The results of each survey are shown in Fig. 1. One reason to explain the lower prevalence rate is the low population age. According to the study done by Bi Y, the floating population is mainly young and middle-aged, with an average age of 33.2 years old [4]. The increase in life expectancy is one of the important factors contributing to the rising prevalence of chronic diseases [9]. Through the analysis of the included literature, we found that the prevalence of diabetes increases with age in the floating population [6, 8, 10–12]. This phenomenon was also found in the survey of other targeted population [13].

### Comparison with the local population

To find the factors that influence the population and summarize the suitable model of disease prevention and control, a comparison of the prevalence rate between the floating population and the local residents is useful. In addition, the analysis of differences on the health consciousness and health service utilization of these two groups provides further evidence. There are no consist findings in the research about the comparison of the prevalence rate between these two groups. In Hainan Province, the prevalence of diabetes in floating population is higher than that of the local residents above 18 years old in five counties in 2010 (6%) and the prevalence rate of people in Meilan District in 2011 (8.2%) [14]. Similar results were found in Wang Wenyun’s study [5]. Researchers find that this can be attributed to that the knowledge and awareness of the prevention and treatment of diabetes in the floating population are lower than those of the local population, while the risk factors such as overweight and obesity are significantly more prevalent than that of the latter. Meanwhile, different studies have obtained different conclusions. According to the study by He Tianjing et al., the prevalence of diabetes, overweight, obesity and other risk factors in floating population in Hubei Province are lower than the local population. The reason for this is that the floating population is relatively younger and mainly occupied with manual work [15]. The inconsistence in results of research can be explained by the demographic characteristics including the aging population, level of education, social economic status differs in different regions of China, and these factors are directly related to the incidence of diabetes.

### The gender difference in diabetes mellitus among floating population

In a nationwide investigation, results show that prevalence rate of the male is higher than that of the female in the population under 50. and the outcome reverse in the population over 60 [10]. The differences are both statistically significant. From a physiological perspective, endogenous sex hormones may play a certain role. The secretion of sex hormone inside human body is directly related to the aging, and the high level of testosterone can increase the risk of type 2 diabetes in women and reduce that in men [16]. According to the study done by Yufang Bi et al., because the floating population is relatively young and the average age of them is 33.2 years old, the prevalence rate of the male is higher than that in the female (male,6.5%,95%CI,6.1–6.8; female,3.3%,95%CI,3.0–3.6) [4]. Similar results can also be found in other studies [6, 8, 11, 12, 17, 18]. It is worth noting that many studies show that the prevalence of diabetes among floating population has no gender differences [5, 9]. For example, Ji Kui et al. show that the difference between the prevalence rate of male and female is not statistically significant (χ2 = 0.66,*P* = 0.42) [19]. Yan Wei’s study in Jiangxi province has drawn the similar conclusion [20].

### The age difference in diabetes mellitus among floating population

Age is an important risk factor for diabetes. In the population of adults over age of 18 in China, the prevalence rate of people aged 18–29 is only 4.5% (95%CI,4.1–5.0). However, the prevalence in the population over the age of seventy has reached 23.5% (95%CI,22.3–24.7) [10]. According to the national survey, although the floating population group is generally young, the result shows that the prevalence increases with age. The prevalence rate of population aged 19–29 years old is 2.1% (95%CI,1.8–2.4), and the prevalence rate of population aged 50–59 years old has reached to 15.8% (95%CI,15.6–18.0). The same results were also found in the regional surveys [6, 8, 11, 12, 14].

### The risk factors of diabetes among floating population

According to the global report on diabetes, physical inactivity and being overweight or obese is strongly linked to diabetes [9]. The relevant domestic survey about floating population rarely had such risk factors included. Through the collation and summary of the literature content, we finally chose the prevalence of overweight and obesity as indicators of risk factors in floating population.

According to “The status of nutrition and chronic diseases in China (2015)” issued by the National Health and Family Planning Commission, the rate of overweight is 30.1% in adults over age of 18, and the obesity rate is about 11.9% [21]. In the floating population, the overweight rate is 26.8%(95%CI,26.4–27.3), and the obesity rate is 4.7%(95%CI,4.5–5.0) [4]. The results of the most regional researches are close to the national level, but there are significant differences in some surveys. In Yin Chuan and Ning Xia, the overweight rate of the floating population is 37.5% and the obesity rate is 26.0% [22]; in Xiang Shan, the obesity rate is 14% [5].Most of these studies didn’t further analyze the reason for the difference of overweight and obesity in the floating population and the general population. The only explanation available is that the floating population are mainly blue collar workers and the front-line workers, mainly engaged in middle or more intensive physical labor, which it can prevent overweight and obesity.

### The disease management of floating population with diabetes

Diabetes is one of the most dangerous risk factors in the world. Excluding the long treatment time and high cost of treatment, if poorly controlled, it is also a risk factor for many other diseases. Research shows that hyperglycemia is the most common cause of chronic kidney disease [23]. In many countries, diabetes is an important reason for blindness in middle-aged population [24]. In addition, latest lot of research shows that hyperglycemia is an important risk factor for different kinds of cancer [25–28]. Simply, it is an important task for the government to treat patients with diabetes for a long time and make their blood glucose levels at an ideal level. The awareness rate, treatment rate and control rate are the important indicators to evaluate a government’s function of prevention and control system.

At present, the awareness rate of diabetes patients in general population is 30.1%(95% CI, 29.1–31.1), the treatment rate is only 25.8% (95%CI, 24.9–26.8), and the control rate is 39.7% (95% CI, 37.6–41.8) [10]. The studies of diabetes among the floating population rarely involved in the disease management. According to the survey by Huang Yingzhe et al., the awareness rate in the floating population with diabetes is 18% [17]. In Sichuan province, the awareness rate of diabetes is 27.8% in floating population. In 2012, the treatment rate is 81.3%, and 40.6% of self-reported diabetes patients participated in the local disease management. At the same time, the disease control rate is 6.5% [19]. In Ning Xia province, the awareness rate is 48% in floating population, the treatment rate is 48%, and control rate is 58.3% [11].In Chongwen District,Beijing, the treatment rate is 89.5% in floating population with diabetes [29].

The current literature shows that there is a big gap between the real situation of disease management in floating population with diabetes and the goal raised by the local health department. And the former is far behind the management level of local population [30]. The main reasons behind the low management rate are as the following. First, because of the onerous work and long working time, the floating people are unable to cooperate with local public health agencies to carry out the relevant public health services. According to the research by Yufang Bi et al., 79.2% (95% CI,78.8–79.7) of those floating people work five days a week, and 39.2% (95% CI, 38.7–39.7) of them work more than 8 h a day. Long time work makes it impossible to receive health education, fasting blood glucose testing and other related services [4]. Second, many floating people irregularly change their jobs, which makes it harder or impossible for the local community health agencies to establish their health records and provide continuous health services. Third, the number of floating population is still increasing [31], which poses a greater challenge to China’s diabetes prevention and control system. There will be a huge demand for basic public health services due to the increasing number of floating population. However, the township hospitals, community health service centers, and other basic health institutions currently lack public health practitioners. In addition, the public health physicians are already very busy to deal with their daily work to treat the local residents, and they have no time to provide extra services to the floating population within the jurisdiction. Fourth, the current allocation mechanism of basic public health service fund is mainly based on the number of registered residents within the jurisdiction, and there is no additional financial support to provide services to the floating people. Without adequate final incentives, basic medical institutions lack the wiliness to give long-term, standardized disease management to the floating population.

## Conclusions

This review includes all domestic research related to floating population with diabetes. As a unique social phenomenon, the floating population is the result of the China’s social development. There is no corresponding sociological concept abroad and no relevant valuable literature.

The main outcome of this review is that the prevalence rate of the diabetes in the floating population in mainland China is lower than that of the general population. In addition, the prevalence of the overweight has no significant difference in both groups. However, the obesity rate in the floating population is lower. Finally, the disease management of the floating population is not able to meet the goal set by the local health department.

The result indicates that the potential threat brought by the diabetes in floating population is imponderable. To achieve the “primary health care for all” health strategic objectives, the relevant departments should take the responsibilities to take care of the vulnerable groups such as the floating population. We should conduct the chronic diseases prevention and intervention work for migrant workers in a planned way, and promote the fairness of society so as to let them play a more prominent role in the process of urbanization.

At present, it is difficult to promote the equalization of basic public health services for the floating population. In order to meet the needs of the floating population with diabetes and the high-risk groups, it is necessary to improve the service delivery system through relevant measures and policies. Recommendations are as follows: 1.establishing a national surveillance system for chronic disease and related risk factor of the population, systematically and periodically gathering the epidemic situation of floating population, developing prevention and control strategies for chronic diseases on the basis of the information, rationally allocating health resources to curb the epidemic risk factors of chronic diseases; 2. Strengthening the health education for floating population in order to enhance the consciousness of active health consultation, improving compliance of the object in the disease management system; at the same time, integrating the existing chronic disease management network, strengthening the ability of the primary medical institutions, cultivating more general practitioners and public health physicians, and improving the ability of public health personnel so that the primary medical institutions are able to provide services to all the population within the jurisdiction; 3. Increasing the fund for public health services on the basis of the currently running mechanism that appropriate expenditure according to the number of registered residence population. Optimizing the existing public health service funding system and taking the floating population into the scope of local public health care system.
